# Stone toolmaking difficulty and the evolution of hominin technological skills

**DOI:** 10.1038/s41598-022-09914-2

**Published:** 2022-04-07

**Authors:** Antoine Muller, Ceri Shipton, Chris Clarkson

**Affiliations:** 1 Computational Archaeology Laboratory, Institute of Archaeology, Hebrew University of Jerusalem, Mount Scopus, Jerusalem, Israel; 2 School of Social Science, University of Queensland, Brisbane, QLD Australia; 3 Institute of Archaeology, Gordon Square, University College London, London, UK; 4 Centre of Excellence for Australian Biodiversity and Heritage, College of Asia and the Pacific, Australian National University, Canberra, Australia; 5 Department of Archaeology, Max Planck Institute for the Science of Human History, Jena, Germany; 6 Australian Research Council Centre of Excellence for Australian Biodiversity and Heritage, University of Wollongong, Wollongong, NSW Australia; 7 Centre for Archaeological Science, School of Earth, Atmospheric and Life Sciences, University of Wollongong, Wollongong, NSW Australia

**Keywords:** Archaeology, Cultural evolution

## Abstract

Stone tools are a manifestation of the complex cognitive and dexterous skills of our hominin ancestors. As such, much research has been devoted to understanding the skill requirements of individual lithic technologies. Yet, comparing skill across different technologies, and thus across the vast timespan of the Palaeolithic, is an elusive goal. We seek to quantify a series of commensurable metrics of knapping skill across four different lithic technologies (discoids, handaxes, Levallois, and prismatic blades). To compare the requisite dexterity, coordination, and care involved in each technology, we analysed video footage and lithic material from a series of replicative knapping experiments to quantify deliberation (strike time), precision (platform area), intricacy (flake size relative to core size), and success (relative blank length). According to these four metrics, discoidal knapping appears to be easiest among the sample. Levallois knapping involved an intricate reduction sequence, but did not require as much motor control as handaxes and especially prismatic blades. Compared with the other Palaeolithic technologies, we conclude that prismatic blade knapping is set apart by being a skill intensive means of producing numerous standardised elongate end-products.

## Introduction

Stone toolmaking has undergone a great deal of technological innovation throughout the last 3.3 million years, resulting in distinctive lithic technologies in different periods. Explaining why a particular technology emerged in a particular period requires an understanding of the innate requirements of each lithic technology. How efficient were certain technologies in terms of production time or raw material cost? How much hierarchical organisation was needed to understand them? How easily did they lend themselves to standardisation? And how difficult were they to knap? Previously, we have attempted to compare the variable levels of efficiency^1^ and hierarchical complexity^2^ involved in different lithic technologies. Here, we focus on the question of difficulty; how much skill do different lithic technologies require for their manufacture? We seek to approximate the requisite skill levels involved in discoidal, handaxe, Levallois, and prismatic blade knapping, which span much of the temporal and geographical history of stone tool making.

For decades, blade production was presumed to represent a dramatic leap in the technological capabilities of past hominins, and even became synonymous with our species^3–5^. In response to this orthodoxy, Bar-Yosef and Kuhn^6^ rightly questioned “the big deal about blades”, and since then there has been a gradual erosion of the traits once thought unique to prismatic blade technology. We now know that systematic blade production far pre-dates the emergence of *Homo sapiens*^7,8^. Prismatic blade technology is not more hierarchically complex than earlier Levallois technology^2^, and blades are not markedly more efficient (at least until pressure blade technology) in terms of the amount of usable edge on all flakes produced compared with other direct percussion technologies of comparable size^1,9,10^. However, the level of skill necessary for prismatic blade knapping in comparison with other technologies has not been previously tested.

The null hypothesis here is that all lithic technologies of the past involve the same amount of skill to manufacture. Of course, we find this null hypothesis to be unlikely, simply by observing the sheer diversity of stone tools made by past hominins in different periods, as well as our own experiences of the difficulties of learning each technology. We can compare for instance, two technologies that approximately bookend the vast swathe of lithic evolutionary history: Oldowan choppers and Neolithic Danish flint daggers. Choppers require only a handful of removals and the final artefact resembles the overall morphology of the original nodule from which it was made. Danish daggers on the other hand, involve a long and complex reduction sequence, requiring multiple percussors and knapping techniques, with a highly stylised end-product entirely unrelated to the shape of the original nodule. We know intuitively that these technologies differ vastly in the level of skill required for their manufacture.

However, the inherent level of skill required for manufacturing other lithic technologies is perhaps less obvious. For instance, does a Levallois or a prismatic blade core require more skill to produce? Levallois reduction requires a long and hierarchically complex sequence of preparatory flake removals, while blade production requires intense platform preparation and a steady and precise hand to deliver blank removal blows. Which requires more skill? There may not be a straightforward answer, but thus far we lack any quantifiable way to compare the skill required for these technologies.

For individual technologies, a wide range of core and flake attributes have been derived archaeologically, ethnographically, and experimentally in order to model skill level. These attributes include a low rate of aberrant flake terminations (step, hinge or overshot), flakes with regular or standardised shapes, flakes that efficiently exploit the length of the core surface, and an absence of ineffective strikes on unsuitably high-angled surfaces or too far from the core’s edge^11–27^.

What is common among these studies is that most seek to reconstruct skill level on individual or site-based scales. Much recent research has been directed towards the process of skill acquisition among novice knappers^28–35^. These studies have revealed a lot about how skill accumulates during the learning process and how variable levels of skill manifest in the archaeological record. To facilitate comparison between the learning trajectories of different knappers, each of these studies investigates skill levels in only a single mode of knapping.

In an attempt to make different stone tool types commensurable, we focus on inherent knapping properties (strike time, platform area, flake size, and relative blank length). By way of example, we can compare again an Oldowan chopper and a Danish dagger. In terms of strike time, knappers of choppers need not invest much time in their striking actions as each strike is accompanied by little risk. By contrast, the fine thinning removals of Danish daggers necessitate much care and deliberation before striking, as each flake removal is crucial for the next removal and for the overall appearance of the final artefact. The flakes removed during chopper and dagger knapping differ markedly too. While the flakes removed from choppers possess large cortical, plain, or dihedral platforms^36,37^, the thinning flakes removed from Danish daggers typically have well-prepared and small platforms^38–41^. The minimum level of strike precision required to successfully remove flakes on Oldowan choppers is obviously less than for the daggers. Similarly, the flakes removed from choppers are proportionally large relative to the core size, while those removed from daggers represent a much smaller fraction of the core, with many times more removed in the more intricate reduction sequence of the latter. To produce more flakes in a reduction sequence requires anticipation of future removals and maintenance of suitable platforms, so this measure may be related to hierarchical complexity. Removing flakes of a desired size and shape has wide margins of error during chopper manufacture, but making dagger thinning flakes of suitable length, without falling short or over-shooting the core surface, requires precise placement, angle, and strength of strikes. The stark difference between these two tool types makes their performance according to these four metrics readily apparent via a thought experiment only. For discoid, handaxe, Levallois, and prismatic blade knapping however, we require replicative experiments to quantify their deliberation (strike time), precision (platform area), intricacy (flake size), and success (relative blank length). These four traits will allow us to compare the requisite skill level for these four technologies.

Skill is comprised of a combination of innate physical and cognitive evolved characteristics, coupled with the accrual of experience via learning and practice^42^. On the evolutionary side of this equation, the development of our hands and brains improved our ability to dexterously manipulate objects such as cores and hammerstones and conceptualise the process of knapping. Throughout our evolutionary history, our hands underwent significant changes, including the development of stronger and longer thumbs, larger joint surfaces, and unique muscle architecture^43–46^. These derived traits accommodated the necessary grip strength and resistance to physical stresses associated with knapping^47,48^, providing hominins with an increasing ability to deftly manipulate hammers and cores. Meanwhile, evolutionary brain enlargement and changes in regions like the prefrontal, temporal and premotor cortices^49–54^ may have enhanced hominin capacities for cognitive traits like working memory, procedural memory, expert cognition, and hierarchical cognition; traits that are all associated with skilful stone toolmaking^2,49,54–62^.

The complex interplay between these physical and cognitive evolutionary traits establishes an upper limit on the level of skill that can be achieved by a hominin knapper. This theoretical maximum can be approached by years of experience. Experience can be accumulated via the interplay between declarative knowledge (*connaissance*) and implicit know-how (*savoir faire*), which are primarily acquired during learning and practice respectively^63,64^. Of course, learning knowledge and practicing know-how are not mutually exclusive, but instead reinforce each other through positive feedback loops, in what has been called the helical curriculum^65,66^. Together, this accrued experience allows knappers to approach the theoretical maximum level of skill determined by their evolutionary constraints.

So far, experimentally disentangling the contribution of evolution and experience has remained difficult, in part because we can observe modern knappers under experimental conditions, but we are of course unable to observe our hominin ancestors. Understandably therefore, most experimental studies of skill acquisition primarily quantify the portion of skill determined by experience, but not the portion determined by evolution. Directly measuring this latter portion of skill is nearly impossible, and not something we attempt here. However, we indirectly incorporate the evolutionary components of skill by approaching the problem from the opposite direction. Instead of observing many knappers of variable skill levels making one technology, we observed one expert knapper making multiple technologies characteristic of different periods.

**Figure 1:**
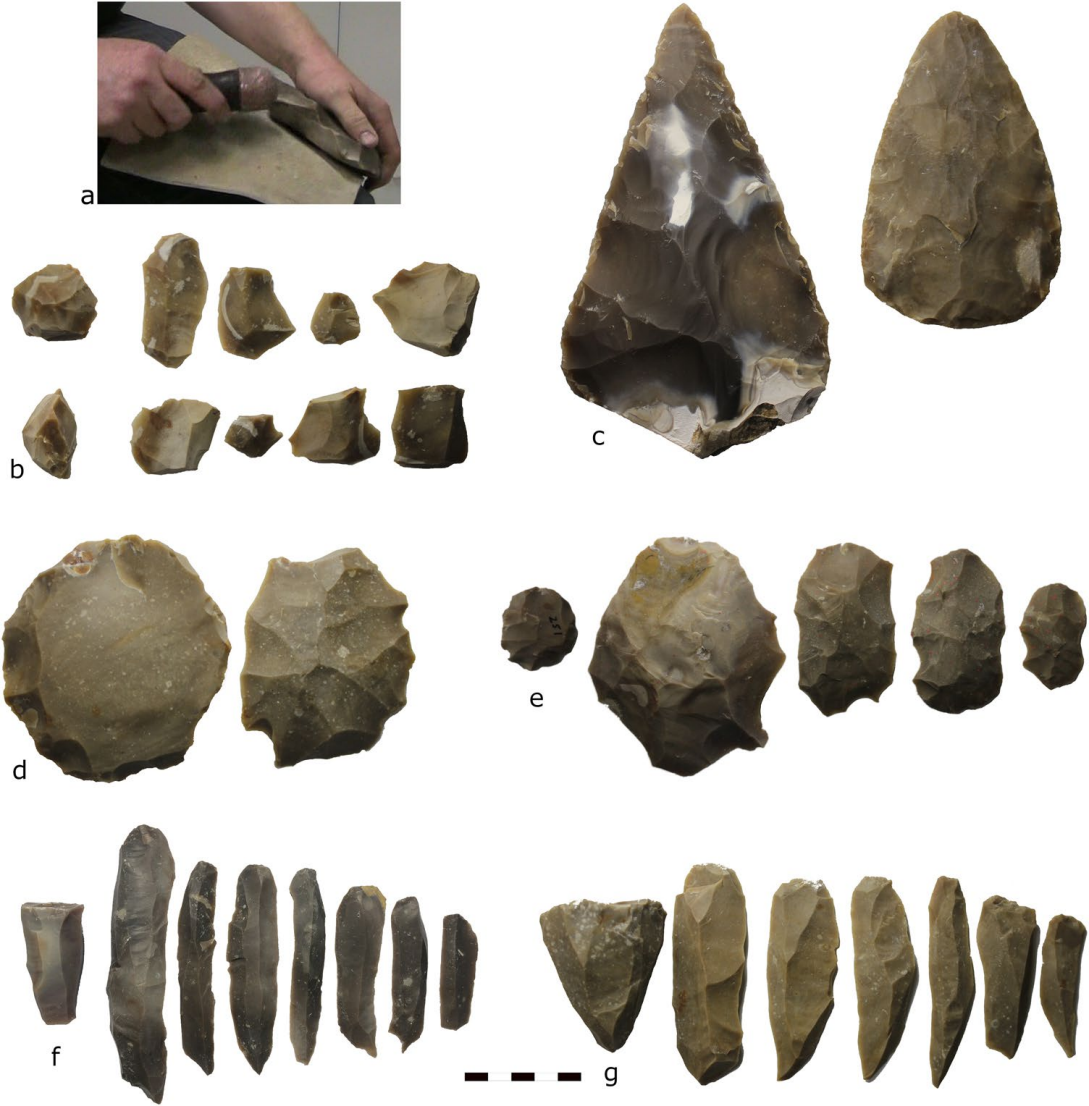
The experimental setup and examples of the lithics.(**a**) Knapping footage. (**b**) Discoidal core and flakes. (**c**) Earlier (left) and later (right) handaxes. (**d**) Preferential Levallois core and flake. (**e**) Recurrent Levallois core and flakes. (**f**) and (**g**) Prismatic blade cores and blades.

We estimate the level of skill required for each technology by quantifying aspects of difficulty for the experimental knapper across discoidal, handaxe, Levallois, and prismatic blade knapping (Fig. 1). Importantly, the experimental knapper is abundantly familiar with all four technologies and has spent a similar amount of time learning and practicing each.

By quantifying difficulty, we are providing a proxy measure for the minimum level of skill required for the manufacture of these technologies, which estimates skill holistically, but blindly. We are unable to disentangle the portion of skill comprised of experience versus the portion comprised of evolutionary factors. However, we are not measuring skill in terms of experience alone, but rather the minimum level of skill necessary for past hominin knappers to successfully achieve a technology; the ‘minimum necessary competence’^67^. Past hominin knappers met this minimum required skill level with some combination of learning, practice, and evolved capacities.

## Results

**Figure 2:**
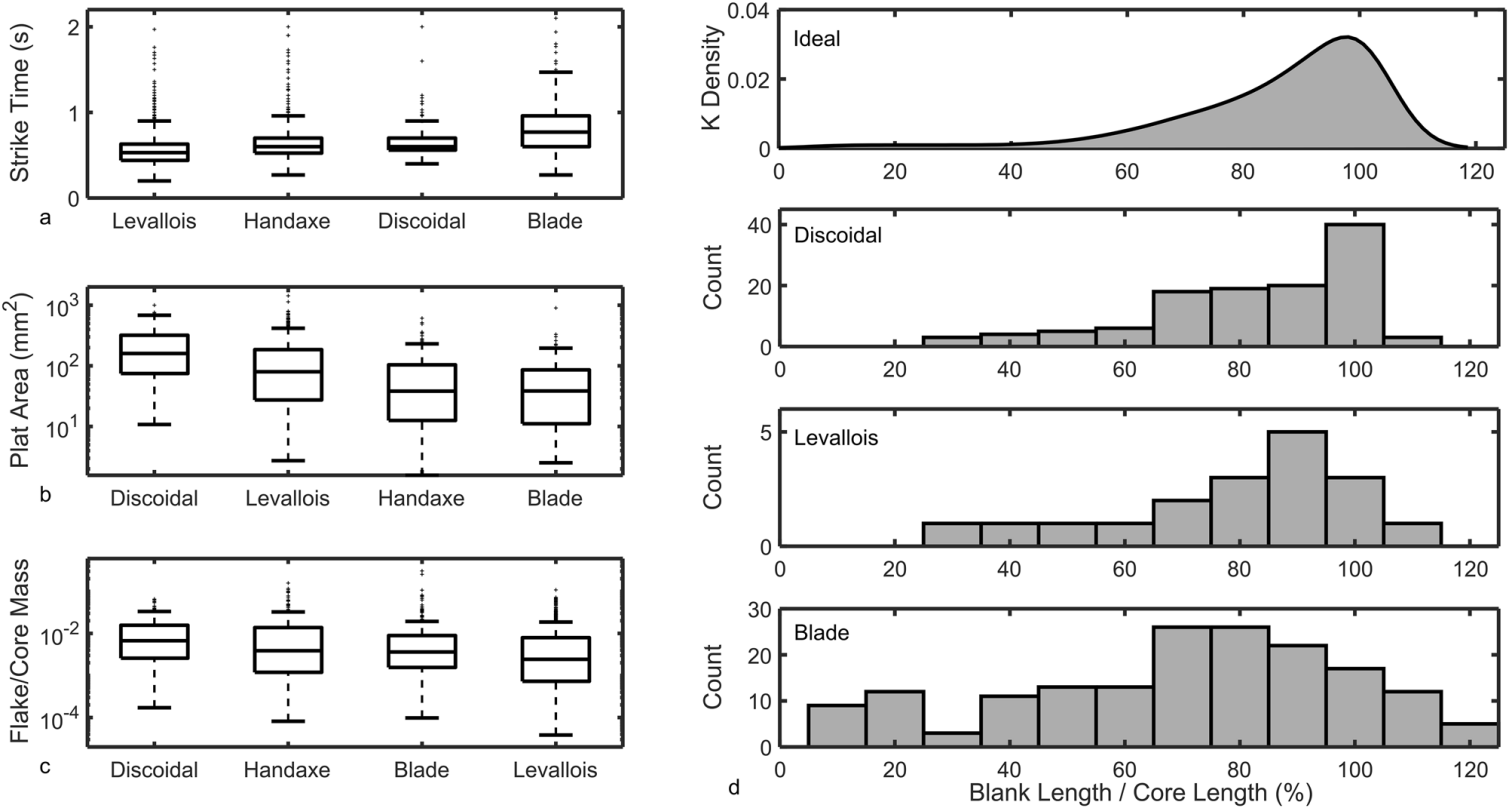
Results of the proxy measures for knapping difficulty. (**a**) Boxplot of the strike times (seconds) taken for Levallois (N = 1757, median = 0.53s), handaxe (N = 649, median = 0.60s), discoidal (N = 250, median = 0.60s), and prismatic blade (N = 469, median = 0.77s) knapping. (**b**) Boxplot of the flake platform area values for the discoidal (N = 150, med = 160.08mm^2^), Levallois (N = 543, med = 79.91mm^2^), handaxe (N = 175, med = 38.27mm^2^), and prismatic blade (N = 184, med = 38.53mm^2^) flakes. (**c**) Boxplot of individual flake mass versus total flake mass from the same core for the discoidal (N = 165, med = 0.0067), handaxe (N = 357, med = 0.0039), prismatic blade (N = 289, med = 0.0036), and Levallois (N = 698, med = 0.0024) iterations of this experiment. A logarithmic scale on the y-axis in **b** and **c** is used to better display the positively skewed data. (**d**) Histograms showing the percent of blank length to core length for the discoidal (N = 118, med = 90), Levallois (N = 18, med = 85) and prismatic blade (N = 169, med = 70) iterations.

The results for deliberation (strike time), precision (platform area), intricacy (flake mass / total mass), and success (blank length / core length) are shown in Fig. 2.

### Deliberation

Figure 2a shows how strike times vary among the different technologies. When a knapper perceives a particular flake removal to be more difficult, they will invest more time in the lining-up and delivery of the strike. Here, all strikes were timed to the nearest tenth of a second, including both successful and unsuccessful strikes (N = 3125). A Mann–Whitney pairwise comparison with a Bonferroni correction for multiple tests reveals that Levallois strikes involved significantly shorter durations than handaxe (U = 400,544, df = 2405, p < 0.001), discoidal (U = 132,177, df = 2006, p < 0.001), and prismatic blade (U = 173,583, df = 2225, p < 0.001) knapping. Meanwhile, flakes removed during the handaxe and discoidal experiments took a statistically equivalent amount of time (U = 75,009, df = 898, p = 0.47). Lastly, prismatic blade knapping involved longer strike times than both handaxe (U = 94,642, df = 1117, p < 0.001) and discoidal (U = 37,339, df = 718, p < 0.001) strikes.

The elongate nature of blade cores may necessitate precise coordination of the platform, flaking surface, and distal end, which requires consideration of the consequences of the strike for the overall form of the piece rather than just the form of a single surface. On blade cores, mistakes are costly, with any repairs to the blank removal surface or platform resulting in significant reduction to the volume of the core.

Another element of knapping that reveals the knapper’s perception of difficulty is how frequently they switch hammers. In the series of experiments designed to test the validity of copper hammers as analogues for natural hammers (see the Supplementary File), the knapper chose from a range of different stone hammers and switched between them freely. Interestingly, Levallois knapping involved the most frequent hammer changes (0.83 switches/min), compared with blades (0.50 switches/min), discoids (0.16 switches/min), and handaxes (0.09 switches/min). This likely reflects the variety of tasks necessitated by Levallois knapping, wherein creating convexities, faceting platforms, and removing Levallois flakes are aided by hammers of different morphologies.

### Precision

Figure 2b shows the strike precision, or the amount of tolerable error in where a core is struck; here measured by platform area. Only flakes with intact platforms are included here (N = 1052). Discoidal platforms were significantly larger than Levallois platforms (U = 27,038, df = 692, p < 0.001), Levallois platforms were significantly larger than both handaxe (U = 34,889, df = 717, p < 0.001) and blade (U = 33,831.5, df = 726, p < 0.001) platforms, while the handaxe and blade platforms were comparable in size (U = 15,278, df = 358, p > 0.99). Handaxe and blade knapping thus require the highest strike precision, followed by Levallois and discoidal knapping respectively. On prismatic cores precise strikes must be applied close to the edge and with the requisite amount of force to travel the length of the core without dissipating early or overshooting.

### Intricacy

Figure 2c shows the intricacy of each technology, quantified by the mass of each flake as a ratio to the total mass of all flakes from the same core. Among the flakes produced during this experiment (N = 1509), those from the discoidal reduction sequences comprised on average a significantly greater proportion of flake mass compared with the flakes removed during the blade (U = 18,560, df = 453, p < 0.001), handaxe (U = 21,370, df = 521, p < 0.001), and Levallois (U = 36,370, df = 862, p < 0.001) iterations. Meanwhile, the flakes removed during blade and handaxe manufacture were statistically comparable (U = 47,410, df = 645, p = 0.46). Lastly, the flakes removed during the Levallois reduction sequences were the smallest of all, being significantly smaller in terms of total flake mass compared with the handaxe (U = 110,800, df = 1054, p = 0.019), blade (U = 81,990, df = 986, p < 0.001), and discoidal (U = 36,370, df = 862, p < 0.001) flakes. In terms of proportional flake mass therefore, Levallois knapping required the most and smallest flakes, making it a more intricate technology to produce.

### Success

Easier lithic technologies should be undertaken more successfully, with flakes travelling across a higher proportion of the core’s length. Figure 2d shows the theoretical ideal distribution of blank lengths compared with the actual blank length proportions for discoidal, Levallois, and prismatic blade knapping. Kolmogorov–Smirnov tests were used to compare the actual distributions of blank lengths with the ideal. Discoidal blanks (D = 0.12, p = 0.38), and to a lesser extent Levallois blanks (D = 0.38, p = 0.074), approximately conform to this ideal distribution, with most blanks between 70–100% of the core’s length. However, the prismatic blade sample includes many blanks that are shorter and longer than ideal, with a significantly flatter distribution (D = 0.32, p = 0.001). Compared with discoidal and Levallois knapping, removing prismatic blade blanks approaching the length of the core is thus more difficult.

A similar pattern emerges when we consider the proportion of different termination types for each technology. Prismatic blade blanks more frequently possess aberrant terminations (27.22%), compared with discoidal (4.24%), and Levallois (11.11%) knapping, as well as a slightly higher breakage rate (23.08% compared with 17.80% and 16.67% for discoids and Levallois respectively). Prismatic blade blanks are significantly more frequently broken or possess an aberrant termination (X^2^ = 24.40, p < 0.001).

For prismatic blades, the long and narrow blanks make successful removals more difficult, as the imparted force from the hammer has more chance to dissipate early, leaving a step or hinge termination. The biconical shape of discoidal cores makes removing relatively large flakes easier, while the lateral and distal convexities of Levallois upper core surfaces assist in making the blank terminate before the edge of the core.

### Stone hammers

As explored in the Experimental Methods section, the Supplementary File contains a methodological test of the suitability of copper hammers as an analogue for natural hammers in this present study. This secondary set of experiments was conducted with the aim of ensuring no confounding influence from this choice of hammers, but it also serves to confirm the key findings of the main portion of this study. Regardless of hammer choice, prismatic blade knapping involved the longest strike times, smallest platform areas, and shortest blank lengths as a proportion of core length, while Levallois knapping involved the most intricate reduction sequences, in terms of relative flake masses (Supplementary File).

## Discussion

Here, we compared for the first time the difficulty of producing four different lithic technologies representing some of the most significant innovations along the branching paths of lithic evolution. We explored how different technologies influence knapping outcomes. We hope future experimentation will incorporate multiple knappers of different skill levels to explore the complex interplay between the technologies, the skill of the knapper, and the final morphology of lithic artefacts produced. Additionally, different technologies could be chosen to explore knapping difficulty, and we hope future studies will examine a greater range of stone tool types made by different knappers. However, we chose these four technologies due to the thresholds they represent in defining periods of the Palaeolithic. By focussing on the more complex technologies of different periods, we can hope to approach an understanding of the technical and cognitive capacities of past hominins. Regardless of which technologies are chosen, the key challenge for quantifying skill lies in finding meaningful comparisons between them.

Discoidal, handaxe, Levallois, and prismatic blade technology possess markedly different goals, reduction sequences, and end-products, making comparisons difficult. We thus sought to identify commensurate traits that could chart the general difficulty involved in their manufacture. Deliberation (strike time), precision (platform area), intricacy (flake mass / total flake mass), and success (blank length / core length) all proved useful at differentiating the technologies. Success (blank length / core length) could not be applied to handaxes, as core-tools do not involve blank removals, but otherwise, the four quantifications of knapping difficulty were applicable to the four technologies. Precision, intricacy, and success primarily relate to how strictly the knapper must apply their strikes in terms of location, angle, and force. The amount of tolerable error in strike position and success has clear implications for the physical components of skill such as manual dexterity and motor control. Meanwhile, strike time and frequency relate primarily to the difficulty level of each strike, as perceived by the knapper, and the overall difficulty of the sequence in terms of the cumulative number of accurate strikes.

Collectively, these metrics revealed the difficulty involved in the four technologies under investigation. Discoidal flaking was repeatedly found to be the easiest among the sample, involving large platforms, large flakes relative to total mass, long blanks relative to flaking surface length, and short strike times.

Levallois technology involved the most intricate reduction sequence, involving the removal of the most and smallest flakes, in accordance with previous analyses showing that it is the most hierarchically complex^2^. However, this long and complicated sequence of flake removals did not require the most attention and deliberation of the knapper. The strike times involved in Levallois knapping were the shortest among the sample, which lends support to previous studies suggesting Levallois draws considerably upon acquired expertise^58,68,69^. The Levallois blanks often approached the length of the core, overshooting only occasionally, showing flaking success was relatively easy to achieve.

Handaxes and prismatic blade cores appeared to require the most motor control. For instance, the platform area of flakes produced during handaxe and blade knapping were the smallest in the sample. Invasive and fragile bifacial thinning flakes require a very precise application of force, close to the core edge, thus resulting in small platforms. Similarly, long blade removals require precise strikes on small, prepared platforms. Beyond precision, blade blanks possessed the shortest length relative to cores, accompanied by the highest frequency of breakages and aberrant terminations. Moreover, in terms of core preparation, blade technology involved the longest strike times.

The null hypothesis that all technologies examined would require the same level of skill can be confidently discarded. Instead, much variability is noted throughout the evolution of lithic technology, with technologies like handaxes and prismatic blade cores requiring a great deal of dexterity and motor control. In terms of the quantifications of difficulty we employed, prismatic blade production was in three out of four cases found to be the most challenging of the technologies investigated.

Why then, at particular times in particular regions, did hominins transition from comparably easier technologies to more difficult ones like handaxes and prismatic blade technology? It is possible that both technologies fulfil functional necessities that offset the greater production costs associated with more skilfully demanding technologies. They may represent different strategies for achieving similar goals. For instance, handaxes provide a long and durable use-edge that can be repeatedly retouched. Similarly, an explanation for the manufacture of blades despite being more skilfully taxing may lie in their elongate and standardised morphology, offering two long and straight cutting-edges. Prismatic blade cores produce many such blanks of predetermined form. From our experiment, of all the flakes produced during Levallois knapping, only 2.58% were predetermined blanks, compared with 40.83% of flakes produced during blade core knapping. For discoidal knapping, 100% of the flakes removed during the sequence can perhaps be considered blanks, but none of these are of predetermined form.

Both Levallois and blade cores involve methods of shaping that influence the morphology of the resultant blanks. With hafting technology likely emerging among Levallois-producing hominins^70–74^, this predetermination may be a response to the need for the predictable end-products that can be attached to a haft. Shifts to blade production may have been driven by the need for greater levels of standardisation, such as when repairing hafted tools or hafting inserts in series^75^. More work is needed to demonstrate the extent to which Levallois blanks and prismatic blade blanks can be standardised. At the very least however, by the Epipalaeolithic and Neolithic, when small, standardised blades were used as serially hafted inserts in tools like sickles, prismatic blade production was meeting this need for multiple standardised blanks^76,77^.

**Figure 3:**
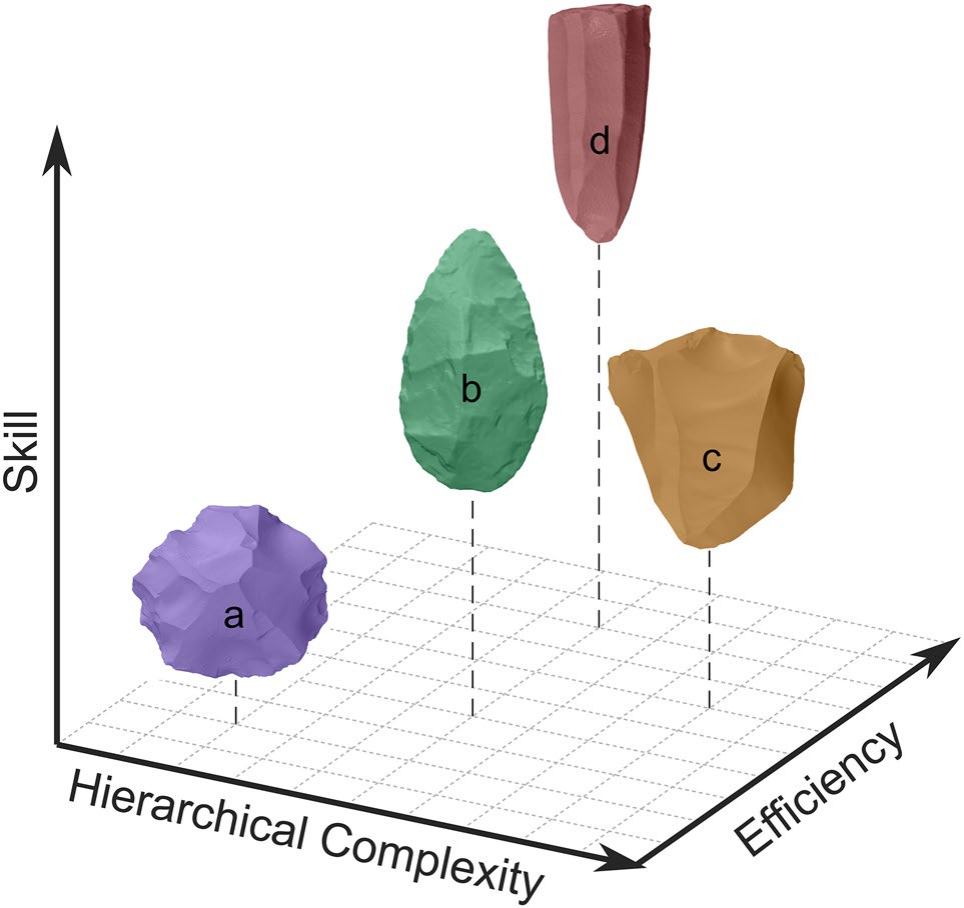
Visual approximation of the inherent traits of the technologies investigated here. 3D scatter plot of the skill, efficiency and hierarchical complexity involved in (**a**) discoidal, (**b**) handaxe, (**c**) Levallois, and (**d**) prismatic blade knapping.

Prismatic blade production was once thought to represent a dramatic leap in the technological complexity of past hominins, but this view has become unpopular in recent decades. However, across almost all proxy measures for knapping difficulty tested here, prismatic blade knapping consistently required the most skill. Collating our findings from other analyses regarding the complexity^2^, efficiency^1^, and skill (present study) of these four different technologies, we can start to encapsulate how these technologies differ in terms of their inherent properties (Fig. 3). Discoidal technology involves lower relative levels of all three traits. Varied levels of complexity and skill can be invested in the production of handaxes, producing many usable flakes and one long, robust use-edge. Meanwhile, Levallois technology requires moderate skill, produces moderate amounts of efficiency, but requires heightened hierarchical complexity. Lastly, prismatic blade technology involves less hierarchical complexity than Levallois, and slightly greater efficiency, but a markedly higher level of skill. Compared with other Palaeolithic technologies therefore, prismatic blade knapping is set apart by being a skilful means of producing a high number of predetermined blanks.

## Methods

As we seek to investigate the level of knapping skill possessed by hominins during different periods of our evolutionary past, it is important that we examine technologies that approach the technological and skilful limits of these periods. For this reason, we chose four technologies that were integral to the history of technological evolution and represent new conceptual leaps and innovations in stone toolmaking: discoidal, handaxe, Levallois, and prismatic blade knapping^78,79^. To ensure experimental control and repeatability, the knapper closely followed known reduction sequences derived both archaeologically and experimentally.

### Sample

Recognised from at least 1.65 mya, and lasting for more than 1.5 million years, handaxes represent a key innovation in the history of lithic evolution. For the experiment, they were knapped according to archaeological and experimental reduction sequences^80–83^, with two equivalent hemispheres maintained via bifacial flaking that exploits the flake scar from the previous strike as the platform for the subsequent strike. The stages of handaxe manufacture identified by Newcomer^81^ were followed by the experimental knapper, namely roughing-out, thinning and shaping, followed by marginal trimming. For the two earlier Acheulean handaxe iterations of the experiment, only minimal thinning and finishing was undertaken to better conform to the larger and slightly cruder handaxes found in the earlier stages of the Acheulean. The shaping stage was aimed at establishing a rounded base and either a pointed or ovate tip, with an axis of symmetry between this tip and base.

Discoidal knapping, a common technology in the Middle Palaeolithic, involved removing flakes bifacially in a centripetal and cordal/tangential pattern, creating a biconical and self-maintaining core^84–86^. Each flake removal leaves a scar which serves as a low angled platform from which further flakes can be removed from the opposing face. For the experiment, the knapper followed Boëda’s^84^ volumetric conception of Middle Palaeolithic discoidal reduction, as well as archaeological^87,88^ and experimental^9,89–91^ accounts of this technology. The goal of discoidal reduction throughout the experiment was to produce as many large, thick flakes from the core as possible while maintaining a biconical and radial core shape. The resultant flakes included the relatively diverse products typical of discoidal reduction, including rectangular, square, and triangular flakes, as well as pseudo-Levallois flakes, and pseudo-blades^9,84,91^. These flakes possessed mostly plain and dihedral platforms^90^. The discoidal reduction conducted in this experiment is closer to that seen in the Middle Palaeolithic than the more ad-hoc and relatively ephemeral appearance of bifacial centripetal flaking patterns occasionally occurring in the Oldowan^21,92,93^.

Following well known reduction sequences^94–97^, preferential and recurrent varieties of Levallois knapping were conducted by establishing a hierarchical plane of intersection between the upper and lower core hemispheres via radial flaking. Unlike discoidal cores, where the interchangeable flaking surfaces are at an angle to the plane of intersection, with increasing convexity as flaking proceeds^84,87,88^, the Levallois flaking surface is approximately parallel to the plane of intersection. Distal and lateral convexities were maintained on the upper hemisphere with shorter dihedral flakes. The platforms for Levallois blank removals were carefully faceted and, when necessary, shaped into *chapeau de gendarme* platforms. Of the five Levallois iterations, one was preferential, two were recurrent centripetal, and two were recurrent point varieties. Levallois flakes were knapped with the aim of removing as much of the upper hemisphere surface as possible, without overshooting the distal or lateral margins.

Lastly, unidirectional prismatic blade knapping involved preparing a strong striking platform, either flat or slightly concave in shape, and removing as many blades as possible from the core face following known reduction sequences^9,98–101^. The striking platform was maintained via faceting and abrasion throughout the reduction sequences. Core tablet (platform rejuvenation) flakes were removed where necessary. The three blade cores knapped for this experiment were all unidirectional, and one involved the use of cresting following archaeological examples^102,103^. While the method was unidirectional, removals from the distal or lateral surfaces of the core were undertaken to maintain the morphology of the flaking surface. Prismatic blades were removed by exploiting long ridges on the core surface, with each removal creating two new ridges along which further blades could be knapped. This generalised method of unidirectional, direct percussion, prismatic blade production was followed throughout the experiment. Prismatic blades have been found since the later Middle Pleistocene^7,8,104,105^, but became more common and formalised in the Upper Palaeolithic^106^.

### Experimental methods

These four lithic technologies were knapped by an experimental knapper (CC) using the same highly cryptocrystalline Texan flint under controlled laboratory conditions. The knapping was filmed, and each flake produced (N = 1509) collected in sequence for later analysis. Every action (strike, core rotation, platform preparation etc.) was timed to the nearest tenth of a second using this footage. Summary data for the 14 reduction sequences that were filmed for this study are shown in the Supplementary Data file.

By using one experimental knapper, experience and evolutionary morphology was kept constant, allowing us to use quantifications of difficulty to estimate the minimum level of skill required for the four different technologies. The experiment was conducted with a view to analysing hierarchical complexity and the resultant data was only reanalysed in retrospect to address the present questions, so the knapper was at the time naïve to the goal and metrics of the experiment.

For all reduction sequences the experimental knapper used the same copper-headed billet as an analogue for natural hammers like those made of antler, wood, bone, or stone. Copper is similar in hardness to natural hammers, while allowing more experimental control. Moreover, we previously found the flakes produced with copper-headed billets to be indistinguishable from those produced with common natural soft-hammers^1^. Copper is obviously anachronistic to the Palaeolithic but was chosen here to maximise experimental control and reproducibility. Actual hammers used in the Palaeolithic are diverse, and the specific hammer used to knap a specific core is very rarely able to be determined. As such, selecting only one natural hammer to use throughout this experiment, or allowing the knapper to use multiple hammer types would introduce much unwanted variability. Moreover, no two natural hammers are the same, in terms of morphology and physical properties, making experiments with machine-made copper hammers considerably more reproducible. If such a hammer breaks during an experiment, it can be exactly replaced. For these reasons, we chose to use a single copper-headed hammer for the primary portion of the experiment. However, to ensure that this choice did not introduce confounding variability, we also conducted a second series of experiments using only stone hammers as a secondary test of this method. The findings of the experiment can only be considered valid if the results obtained using a copper hammer are similar to those obtained with the stone hammers.

See the Supplementary File for the results of this methodological test. Importantly, every measure considered in this study performed similarly under the two test conditions: copper hammer and stone hammers. Thus. for this experiment at least, the use of a copper hammer does not add confounding influence to the results. Of course, for more replicative experiments aimed at reconstructing past knapping methods and reduction sequences, we caution against the use of copper hammers. But we find them suitable for lithic experiments conducted under laboratory conditions requiring tight experimental control. This is in line with much modern lithic experimentation, where more control necessitates more anachronism. For example, the most controlled lithic experiments are those conducted with ball bearings or pistons on glass cores^107–110^. None of these items are present in prehistory, yet these experiments have revealed much about fracture mechanics and past human behaviour.

The data for this present study centres on the timed footage and measured flakes produced during the primary 14 repetitions of the experiment (not the 4 repetitions from the method validation study conducted with stone hammers). More iterations of each technology were conducted for those that possess a greater number of variants. For instance, five Levallois reduction sequences were knapped to encompass preferential, recurrent, and point variants of this technology. Meanwhile two handaxes were produced using simple shaping techniques that resemble those in the earlier parts of the Acheulean, and two were produced using complex thinning techniques, such as turning the edge and platform preparation, that resemble some in the later parts of the Acheulean. See the Supplementary Data file for a summary table of these 14 iterations of the experiment.

Despite the wide range of technologies and variations under investigation, they all involve different combinations of the same physical gestures or actions, and all involve the creation of flakes. Due to these shared qualities, exploring the time taken for particular knapping actions and the morphology of the resultant flakes allows us to compare these otherwise markedly different technologies. We therefore seek to identify metrics of these actions and flakes that quantify knapping difficulty, which are also shared among the different technologies.

### Deliberation

Whether consciously, or subconsciously, a knapper must choose how much time they invest into an individual flake removal. A knapper is likely to take great care and spend more time ‘lining-up’ a strike when it is more difficult to successfully remove. Shorter strike times occur when difficulty is low, and the flaking can be approached more haphazardly.

During these experiments, every action involved in knapping was timed to the nearest tenth of a second using the video player timestamp, including each individual strike against the core. This allows us to compare the strike time for each of the four technologies under investigation here, which is the duration between the moment the hammer is placed near the point on the core where it is to be struck, to the moment the flake is removed by the strike. This includes any small motions that withdraw the hammer from the core in order to ‘line-up’ a strike, as well as the time taken to draw-back the hammer-holding hand prior to the blow, and finally the time taken to deliver the blow.

In a sense, this is a phenomenological measure of how much the knapper deems a strike to be important and difficult. It is thus a subjective measure when comparing different knappers, but internally consistent when the strike times of only one knapper are compared, as was done here. In this way, strike time reveals much about how knappers apportion their time and about the difficulty level they personally perceive.

### Precision

A key component of knapping skill is the dexterous manipulation of hammers and cores. The ability to strike a core’s platform precisely with sufficient force is the difference between a successful flake removal and a flake with an aberrant termination or no removal at all. Strike precision has been used to quantify knapper skill before, with knappers of different skill levels asked to mark their intended impact point on the core^15,29^. The difference between the intended and actual impact point provides a useful metric for knapper strike precision, with skilled knappers striking close to their intended mark.

Rather than focussing on the difference among knappers of different skill levels, here we are interested in the inherent differences among various technologies. By virtue of their core morphology and associated fracture mechanics, flakes struck with great precision close to the platform edge result in small platforms. Even a small deviation from that actual impact point would have resulted in a vastly different removal, or even more likely, no removal at all. Meanwhile, for flakes with large platforms, a similarly successful flake could have been removed even if the knapper struck the core a few centimetres away from the actual impact point.

Platform area (platform width x platform thickness) is therefore a proxy measure for the amount of tolerable error in strike precision. Technologies that routinely produce flakes with smaller platforms allow for less error and thus require more precision. We are not measuring the precision of strikes for a particular flake (a trait inherent to the knapper), but rather we are measuring the minimum required amount of striking precision (a trait inherent to each of the technologies).

### Intricacy

More difficult and complicated technologies should involve a larger number of small flakes. The mass of each flake removal as a proportion of the total mass of all flakes removed from the core offers a quantification of knapping intricacy. Mass was recorded with digital scales to the nearest hundredth of a gram.

Technologies that require more flakes and smaller flakes are inherently more difficult, as to sustain suitable platforms for many removals, blows to the core must be precise and appropriately angled and imparted within a narrow range of force. By contrast, larger flakes allow greater leeway in these variables, and are therefore more forgiving of the knapping errors that occur frequently among less skilled knappers.

### Success

To remove a flake of a desired size and shape requires careful control of strike placement, angle, and force. The efficiency of core surface exploitation has previously been used by Eren et al.^24^ to chart skill acquisition during the Levallois learning process. They used the proportion of flake surface area to core ventral surface area as a measure of how well the knappers established their core morphology and controlled the placement, angle, and force of their final strike.

To make comparisons between technologies easier, we compared the proportion of each blank removal’s length to the length of the core at the point of its removal. Here, we make a distinction between blank removals and preparatory removals. Preparatory removals are flakes that serve to shape the core for later blank removals, while blank removals are the desired flakes removed from a core with the intention to either use as an unretouched tool, or to retouch into a specific tool type. Blanks must also satisfy typological constraints. For instance, blade blanks must be at least twice as long as they are wide, with parallel or convergent lateral margins. The handaxe iterations of the experiment were excluded from this metric as they are not primarily made to create blanks. Of course, the flakes involved in producing handaxes could be used as blanks^111^, but most agree that their primary function is the thinning and shaping of core-tools.

We assume that for discoidal, Levallois, and prismatic blade knapping, an efficient and desired blank removal will as closely as possible approach 100% of the core’s length, without overshooting. For Levallois and blade cores this core length is measured orthogonally from the platform surface to the end of the core, while for discoids, the maximum core length is measured from the intersection of the hemispheres where the flake was struck to the central apex of the core’s biconical morphology. The ratio of blank length to core length could not be physically measured, as that would involve interrupting the knapping process and potentially influencing the natural decision-making process of the knapper. Instead, we used the knapping footage to visually estimate the proportion of blank to core length in increments of 10%. As each blank was removed, the length of its negative scar was compared to the length of the core at the time of blank removal.

Ideally, blanks should mostly be around 70–100% of the core’s length. Blanks less than 70% have consumed a viable platform without exploiting much of the core’s surface. Conversely, blanks greater than 100% have overshot, both shortening the core’s flaking surface and resulting in a flake with a bulky and dull termination. Technologies for which it is less difficult to efficiently remove flakes should have very few overshot blanks.

## Supplementary Information

Supplementary Information 1.

Supplementary Information 2.

## Data Availability

All data generated or analysed during this study are included in this published article (and its supplementary information files).
