# Supplementary material for: Stone toolmaking difficulty and the evolution of hominin technological skills
Source: Sci Rep. 2022 Apr 7;12:5883. doi: 10.1038/s41598-022-09914-2 (PMC8989887; doi:10.1038/s41598-022-09914-2)
Supplement: Supplementary file 2 — Supplementary Information 2. [file 41598_2022_9914_MOESM2_ESM.docx]

**Title:** Stone toolmaking difficulty and the evolution of hominin technological skills

**Authors:** Antoine Muller, Ceri Shipton, Chris Clarkson

**Supplementary File:** Exploring the role of hammer type on knapping difficulty

To test if copper adds confounding variability to the four metrics tested in the manuscript we conducted a methodological validation study, comparing the metrics explored in the manuscript under two conditions: copper hammer versus stone hammers. In addition to the 14 reduction sequences conducted with a copper hammer forming the primary data for this study, we conducted an additional 4 reduction sequences, one for each technology, using stone hammers instead. Throughout these 4 iterations, the knapper was free to select from a number of different stone hammers.


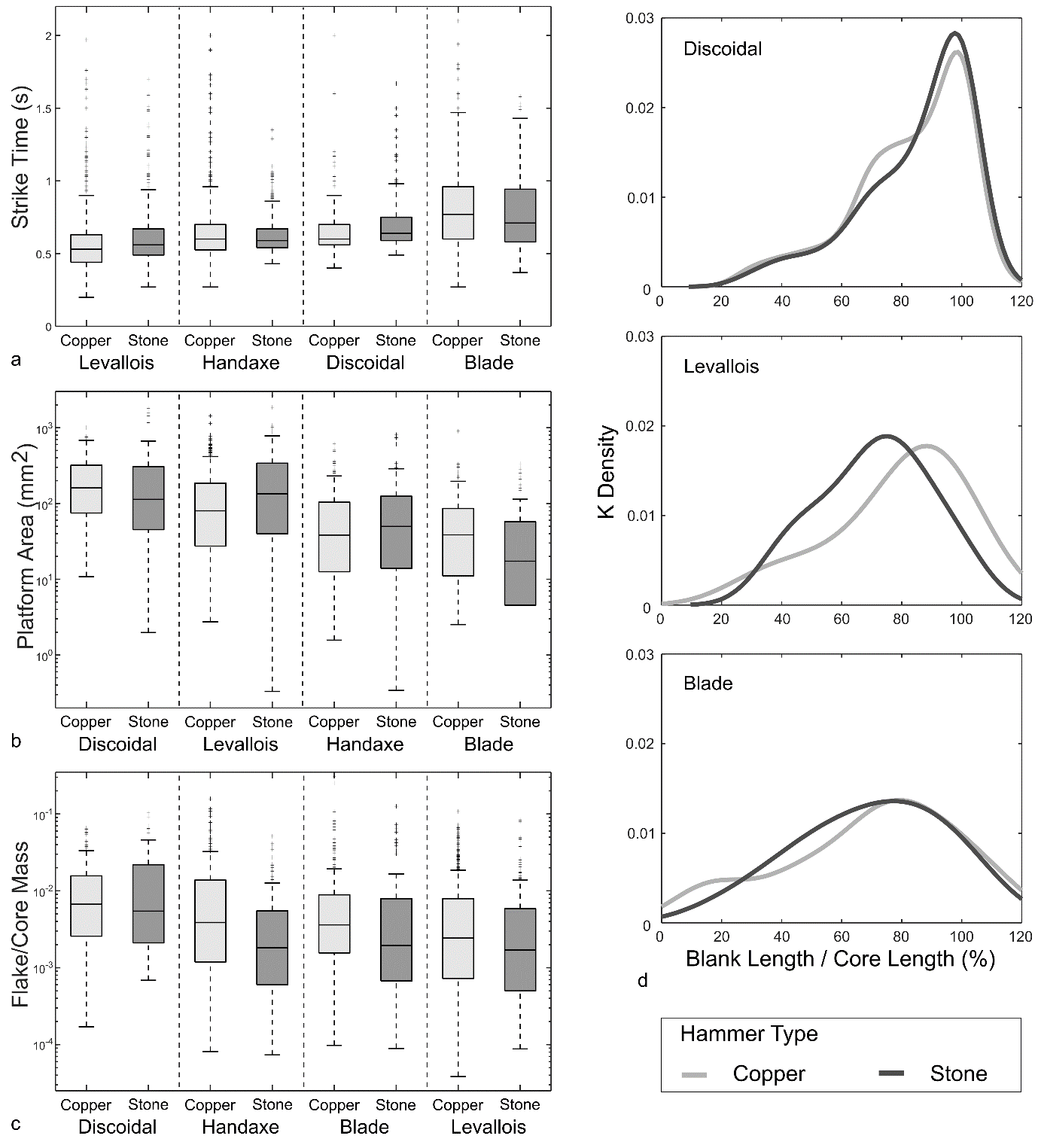


**Figure S1. Results of the methodological validation study testing the role of copper hammers.**

a: Boxplot of strike time. b: Boxplot of platform area. c: Boxplot of individual flake mass versus total flake mass from the same core. d: Kernel density plots of blank length as a proportion of core length for the discoidal, Levallois, and prismatic blade core iterations of the experiment. Note the log10 y-axes in **b** and **c**.

From these results, it is clear that much greater variability exists between the technologies, than between the hammer types. Importantly also, the pattern of results is remarkably similar. For instance, blades have the longest strike times and smallest platform areas compared with the other technologies regardless of hammer type. In terms of every measure considered in this study, the findings of the primary experiment (using a copper hammer) conform to the same pattern as the results obtained from this methodological validation study (using stone hammers). For the purposes of this experiment, and in the interests of repeatability and experimental control, we find copper hammers a suitable analogue for natural hammers.
